# Silencing of Receptor Tyrosine Kinase ROR1 Inhibits Tumor-Cell Proliferation via PI3K/AKT/mTOR Signaling Pathway in Lung Adenocarcinoma

**DOI:** 10.1371/journal.pone.0127092

**Published:** 2015-05-15

**Authors:** Yanchun Liu, Hui Yang, Tianxing Chen, Yongbin Luo, Zheyuan Xu, Ying Li, Jiahui Yang

**Affiliations:** 1 State Key Laboratory of Phytochemistry and Plant Resources in West China, Kunming Institute of Botany, Chinese Academy of Sciences, Kunming, Yunnan, China; 2 Department of Pathology, The First People’s Hospital of Yunnan Province, Kunming, Yunnan, China; 3 Department of Thoracic Surgery, The First People’s Hospital of Yunnan Province, Kunming, Yunnan, China; Cellcuity, UNITED STATES

## Abstract

Receptor tyrosine kinase ROR1, an embryonic protein involved in organogenesis, is expressed in certain hematological malignancies and solid tumors, but is generally absent in adult tissues. This makes the protein an ideal drug target for cancer therapy. In order to assess the suitability of ROR1 as a cell surface antigen for targeted therapy of lung adenocarcinoma, we carried out a comprehensive analysis of ROR1 protein expression in human lung adenocarcinoma tissues and cell lines. Our data show that ROR1 protein is selectively expressed on lung adenocarcinoma cells, but do not support the hypothesis that expression levels of ROR1 are associated with aggressive disease. However silencing of ROR1 via siRNA treatment significantly down-regulates the activity of the PI3K/AKT/mTOR signaling pathway. This is associated with significant apoptosis and anti-proliferation of tumor cells. We found ROR1 protein expressed in lung adenocarcinoma but almost absent in tumor-adjacent tissues of the patients. The finding of ROR1-mediated proliferation signals in both tyrosine kinase inhibitor (TKI)-sensitive and -resistant tumor cells provides encouragement to develop ROR1-directed targeted therapy in lung adenocarcinoma, especially those with TKI resistance.

## Introduction

Lung adenocarcinoma is the most frequent type of lung cancer and the most common cause of death from cancer [1–3]. The poor prognosis of lung cancer patients depends in part on the relatively low sensitivity of lung cancer to chemotherapy. Until recently, first-line therapy in metastatic non-small cell lung cancer (NSCLC) was based on platinum-based doublet chemotherapy [4]. The development of inhibitors targeting the HER family, particularly HER1 or epidermal growth factor receptor (EGFR), has revolutionized therapy [5–8]. These agents are particularly effective in NSCLC patients harboring sensitizing EGFR mutations [9]. Many more inhibitors of specific driver mutations involving genes like ROS, c-MET, FGFR, mTOR, IGFR and RET are currently under development [10–14]. However, efforts to target some mutated genes like K-RAS have been unsuccessful. Moreover, the emerging challenge of acquired resistance to initially effective therapy is becoming another major concern. It has been reported that 10% of patients present with primary tyrosine kinase inhibitor (TKI) resistance, and 50% of the others develop secondary resistance within 9–12 months after starting TKI therapy [15]. Clearly development and application of new therapeutic strategies is essential to improve the prognosis of this disease.

The receptor tyrosine kinase-like orphan receptor 1 (ROR1) is an embryonic glycoprotein involved in differentiation, proliferation, migration and survival during development. ROR1 belongs to the ROR receptor tyrosine kinase family, where the only other known member is ROR2, which shares 58% amino acid sequence identity. The structure of human ROR1/2 consists of an extracellular immunoglobulin-like (Ig) domain at the amino terminus, followed by a cysteine-rich domain known as a frizzled domain, and then a kringle domain [16,17]. After embryonic development, ROR1 is largely down regulated in human cells, but a low level of ROR1 expression is seen in adipose tissue and to a lesser degree in the pancreas, lung, and a subset of intermediate B cells [18,19]. In previous studies, we and others found that ROR1 was expressed by numerous blood and some solid malignancies, including lung cancer cell lines and tissues [20–31]. However, it was not known whether ROR1 expression in patients with lung adenocarcinoma had functional and clinical significance. In the current study, we carried out a comprehensive analysis of ROR1 protein expression in human lung adenocarcinoma tissues and cell lines and found that ROR1 protein is selectively expressed on lung adenocarcinoma, but almost absent from tumor-adjacent tissues and propose that ROR1 could mediate cell survival via the PI3K/AKT/mTOR signaling pathway.

## Materials and Methods

### Cell lines, Tissues, and Cell culture

The NSCLC cell lines MSTO-211H, NCI-H1975, and NCI-H358 were purchased from Typical Culture Preservation Commission Cell Bank, Chinese Academy of Sciences. A549 was kindly gifted by Dr. Guangzhi Zeng of Kunming Institute of Botany, Chinese Academy of Sciences [32]. We received kind gifts of XLA-07,which is a human lung adenocarcinoma cell line from a female patient in China [33], from Professor Yong Duan of First Affiliated Hospital of Kunming Medical University and of PC9 from Dr. Jun Zhang of Shanghai Pulmonary Hospital [34]. A total of 37 paraffin-embedded tissue samples of lung adenocarcinoma patients were collected from First People’s Hospital of Yunnan Province. The study was approved by the Hospital Ethics Committee (No. 2014WL001), and appropriate written informed consent was obtained from each patient. None of the patients received therapy prior to collecting the tissue samples. The diagnostic and TNM stage criteria (extent of tumor, spread to lymph nodes and metastasis) of NSCLC were according to recommendations from the American Joint Committee on Cancer (AJCC) and the International Union against Cancer (UICC). All cell lines were cultured at 37°C in a 5% CO_2_/95% humidified air incubator (Thermo Scientific, Rockford, IL USA) in RPMI-1640 (HyClone, Logan, UT, USA) supplemented with 10% fetal bovine serum (FBS, BI, Kibbutz, Israel) and 100 μg/ml penicillin/streptomycin (BI, Kibbutz, Israel).

### Flow Cytometry Analysis for ROR1 Expression

Cells were stained using standard flow cytometry methodology. Briefly, 5×10^5^ cells were collected and washed twice with ice-cold flow cytometry buffer (PBS with 1% FBS). Five μg/ml of polyclonal goat anti-ROR1 antibody (R&D system, Minneapolis, MN, USA) or chimeric rabbit/human anti-ROR1 monoclonal antibody R12 with HA tag which was developed by the correspondence author in Christoph Rader’s lab [21], or normal goat IgG (Santa Cruz Biotechnology, Dallas, TX, USA) was added to the cells and incubated on ice for 30 min before washing twice with flow cytometry buffer. Biotin conjugated donkey anti-goat IgG (R&D system, Minneapolis, MN, USA) or PE-conjugated anti-HA monoclonal Ab (mAb) (Miltenyi Biotech, San Diego, CA,USA) was added and incubated on ice for 30 min. After washing twice with flow cytometry buffer, R-phycoerythrin conjugated streptavidin (R-PE-Streptavidin, Southern Biotech, Birmingham, AL, USA) was added and incubated on ice for 20 min. Finally, cells were washed with flow cytometry buffer and suspended in 500 μl of flow cytometry buffer supplemented with 5 μl of propidium Iodide (PI, BD Biosciences, Mountain View, CA, USA). A FACSCalibur flow cytometer (BD Bioscience, Mountain View, CA, USA) was used to analyze ROR1 expressing cells and the data were analyzed using the FlowJo 7.6.2 software program.

### Immunohistochemical Analysis

ROR1 was detected in formalin-fixed, paraffin-embedded tumor tissues. Briefly, sections were incubated with rabbit anti-ROR1 polyclonal antibody (pAb) (EPITOMICS, Burlingame, CA, USA) (dilution 1:100) or normal rabbit IgG (Santa Cruz Biotechnology, Dallas, TX, USA) overnight at 4°C. After washing, sections were incubated with peroxidase-labeled affinity purified antibody to rabbit IgG (H+L) (dilution 1: 50; KPL, Gaithersburg, MD, USA) for 30 minutes at room temperature (RT). The reaction product was visualized with the prepared liquid DAB^+^ substrate-chromogen solution (Dako Denmark A/S, Glostrup, Denmark) for 5 minutes at RT. Finally, sections were counterstained with hematoxylin. The immunoreactivity was evaluated independently by two experienced pathologists. The expression level of ROR1 was evaluated by a semi-quantitative scoring system (0–3) as follows: A score of 0 indicates that none of the cells within the sample bound to the anti-ROR1 pAb; a score of 1 indicates low-level binding of the pAb on more than 25% of tumor cells; a score of 2 indicates low-level binding of the pAb on more than 50% of tumor cells or moderate-level staining on more than 25% of tumor cells; a score of 3 indicates moderate-level staining on more than 75% of tumor cells or high level staining on more than 50% of tumor cells.

### Silencing of Human ROR1

The pre-designed siRNA sequences used to target endogenous ROR1 mRNA were obtained from Life Technologies (Grand Island, NY, USA): ROR1 siRNA #1 (siROR1 #1) sense 5’-GUACUGCGAUGAAACUUCATT-3’; antisense 5’-UGAAGUUUCAUCGCAGUACGG-3’ or synthesized from Sigma-Aldrich (Saint Louis, MO, USA): siROR1 #2 sense 5’-CAGCAATGGATGGAATTTCAA-3’; antisense 5’-UUGAAAUUCCAUCCAUUGCUG-3’. Negative control siRNA (siControl, Sigma-Aldrich, Saint Louis, MO, USA) was used as a negative control. Cells were seeded in 96-well plates at a density of 5×10^3^ cells/well and incubated for 12 h in a CO_2_ incubator. Cells were transfected with 10 nM siROR1 or siControl and then serum-starved for 12 h. All siRNA transfections were performed in serum-free medium using lipofectaimine RNAiMAX (Life Technologies, Grand Island, NY, USA) according to the manufacturer’s instruction. Cells were harvested for flow cytometry assay at three days or MTS cytotoxicity assay at five days after siRNA transfection.

### MTS Cytotoxicity Assay

Cells cultured in complete medium supplemented with 10% FBS (BI, Kibbutz, Israel) were plated in 96-well plates (5×10^3^ cells/well). Erlotinib, Gefitinib or siRNA was added at 24 h or 12 h, respectively. After 3–5 days, cell cytotoxicity was performed using the CellTiter 96 AQueous One Solution Reagent (Promega, Madison, WI, USA) according to manufacturer’s instructions. Briefly, 20 μl of CellTiter 96 AQueous One Solution Reagent was added into each well of the 96-well assay plate and the cells were further incubated at 37°C for 1 h. Cell viability was then measured by reading the absorbance at 490 nm with a microplate reader (Bio-Rad, Hercules, CA, USA). Experiments were performed in triplicate. The following formula was used to determine cell growth ratio values: 100* (A490 (sample, T)-A490 (sample, T0)) /(A490 (control, T)-A490 (control, T0)).

### IC_50_ Determination of Erlotinib and Gefitinib

Cells were seeded in 96-well plates at a density of 5×10^3^ cells/well and incubated at 37°C for 24 h in a humidified CO_2_ incubator. Cells were treated with Erlotinib or Gefitinib (Cayman, Ann Arbor, Michigan, USA) with doses ranging from 0.005 μM to 50 μM for 72 h. Then, the MTS cytotoxicity assay was used to analyze the IC_50_ value.

### Western Blot Analysis

NCI-H1975 and PC9 cells (5×10^5^) were transfected with 10 nM siROR1 or siControl and then serum-starved for 12 h. At 72 h after siRNA transfection, cells were lysed with 300 μl of lysis buffer (Beyotime, Shanghai, China). All protein extraction buffers were supplemented with protease inhibitor cocktail (Millipore, Bedford, MA, USA) and phosphatase inhibitor (Roche, Basel, Switzerland). The protein concentration of each cell lysate was determined using the Enhanced BCA Protein Assay Kit (Beyotime, Shanghai, China). Normalized amounts of the lysate samples were loaded and electrophoresed on SDS-PAGE gels and transferred to polyvinylidene difluoride (PVDF) membranes (Millipore, Bedford, MA, USA). Immunoblotting was performed using antibodies detecting mTOR, phospho-mTOR, PTEN, phospho-PTEN, AKT, phosphor-AKT (Cell Signaling Technology, Danvers, MA, USA) with β-actin (TransGen, Beijing, China) used as loading control. HRP conjugated anti-mouse IgG (TransGen, Beijing, China) or anti-rabbit IgG (Abcam, Cambridge, UK) was used as secondary antibodies. An enhanced Pierce ECL Western Blotting Substrate (Thermo Scientific, Pittsburgh, PA, USA) was used to detect chemiluminescence.

### Apoptosis Assay

Cell apoptosis was evaluated by flow cytometry analysis. Cells were seeded in 6-well plates at 1.5×10^5^ cells/well. After 12 h, medium was replaced and cells were transfected with 10 nM siROR1 or siControl and then serum-starved for 12 h. After 96 h of incubation at 37°C in humidified air with 5% CO_2_, cells were collected, washed twice with PBS and then resuspended in 100 μl of 1×binding buffer at a concentration of 1×10^6^ cells/ml. Five μl of FITC-conjugated Annexin-V and PI (BD Biosciences, Mountain View, CA, USA) were added to the cells and incubated in the dark for 15 min at RT. 1×binding buffer (400 μl) was added to each tube and apoptosis was measured by flow cytometry (FACSCalibur, BD Biosciences).

### Statistical Analyses

Data are presented as the mean ± S.E.M for the indicated numbers of independently performed experiments. A two-tailed Student t-test was used to determine statistical differences in levels of cell proliferation. Mann-Whitney U test was used to evaluate the ROR1 expression in different gender. All analyses were performed using GraphPad Prism version 6 (GraphPad Software Inc, San Diego, CA, United States) and SPSS 16.0 statistic software (SPSS Inc, Chicago, IL). Differences were considered to be statistically significant when p < 0.05.

## Results

### ROR1 Protein Was Uniformly Expressed by Human Lung Adenocarcinoma

**Fig 1:**
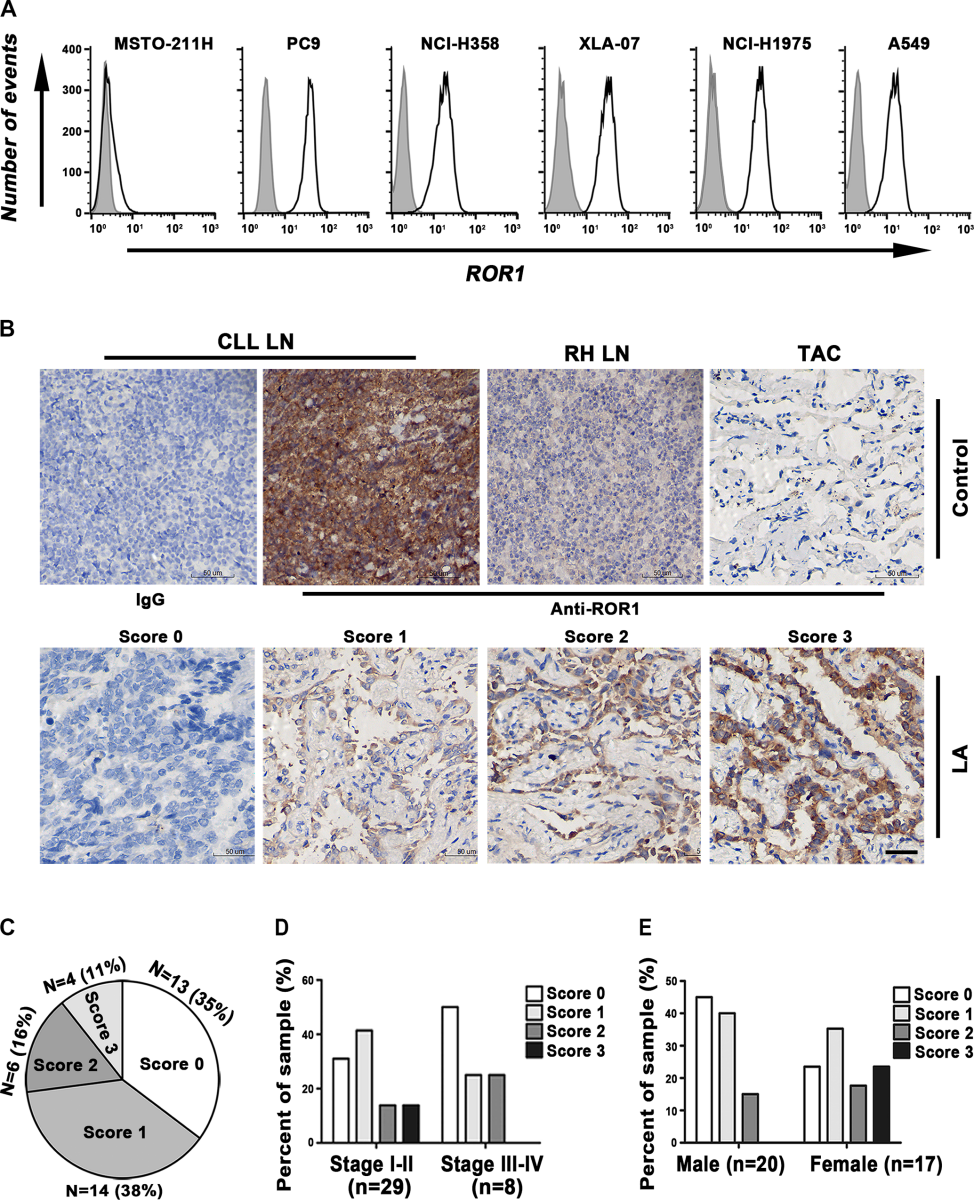
ROR1 expression in lung adenocarcinoma. (A) Flow cytometry analysis of ROR1 protein expression on the surface of NSCLC cell lines. The histograms on the right represent staining for ROR1 with polyclonal goat anti-ROR1 antibody. The background signal with normal goat IgG is shown in gray shadow. (B) Representative images of lung adenocarcinoma tissues (LA) detected by immunohistochemical analysis. Formalin-fixed, paraffin-embedded tissues were stained with polyclonal rabbit anti-ROR1 antibody or normal rabbit IgG. Tissue-bound ROR1 is shown in brown and the nucleus counterstained with hematoxylin is in blue (scale bar in the bottom right picture represents 50μm). A score of 0 indicates that none of the cells within the sample bound to the anti-ROR1 pAb; a score of 1 indicates low-level binding of the pAb on more than 25% of tumor cells; a score of 2 indicates low-level binding of the pAb on more than 50% of tumor cells or moderate-level staining on more than 25% of tumor cells; a score of 3 indicates moderate-level staining on more than 75% of tumor cells or high level staining on more than 50% of tumor cells. Controls include: Chronic lymphocytic leukemia lymph node (CLL LN) stained with normal rabbit IgG as negative control and with polyclonal rabbit anti-ROR1 antibody as positive control; Reactive hyperplasia of lymph node (RH LN) and tissues judged to be adjacent to cancer (TAC) stained with polyclonal rabbit anti-ROR1 antibody as negative controls. (C) A summary of immunohistochemical analysis for ROR1 staining in lung adenocarcinoma specimens. The proportion of lung tumor tissues found negative (Score 0) or having weak (Score 1), moderate (Score 2) or strong staining (Score 3) for ROR1 is indicated in the pie chart. (D-E) The proportion of lung adenocarcinoma tissues of different stages or sex found lacking staining (Score 0) or having weak (Score 1), moderate (Score 2) or strong staining (Score 3) for ROR1 is indicated in each bar. The number of different cases examined for each group is indicated in the parentheses. Statistical analyses was performed using Mann–Whitney U test. p = 0.048.

To examine expression of ROR1 in lung adenocarcinoma, we first chose Erlotinib-sensitive cell line PC-9, Erlotinib-partially sensitive cell line NCI-H358, and Erlotinib-resistant cell lines XLA-07, A549, NCI-H1975, and MSTO-211H (Table 1), to determine ROR1 expression by flow cytometry (Fig 1A). We found that except for MSTO-211H, all the cell lines examined expressed surface ROR1 uniformly, with PC-9, NCI-H1975, and XLA-07 exhibiting the highest expression (ROR1 mean fluorescence intensity (MFI), 37.06, 32.74, and 30.94, respectively). ROR1 expression was not associated with Erlotinib sensitivity, because it was expressed on both Erlotinib-sensitive and-resistant cell lines. In the following experiment, we chose MSTO-211H as ROR1 negative cell line, PC-9, XLA-07, and NCI-H1975 as ROR1 positive cell lines to determine the role of ROR1 in lung adenocarcinoma. In tissues of lung adenocarcinoma patients we found that totally 65% (24/37) of human lung adenocarcinoma expressed ROR1, including 38% (14/37) with weak or focal expression (Score 1), 16% (6/37) with moderate expression (Score 2), and 11% (4/37) with strong expression (Score 3) (Fig 1C). Additionally, among lung adenocarcinoma patients with stage I and II (n = 29), 41% (12/29) had weak or focal expression, 14% (4/29) had moderate expression, and 14% (4/29) had strong ROR1 expression. Among lung adenocarcinoma patients with stage III and IV (n = 8), 25% (2/8) had weak or focal expression, 25% (2/8) had moderate expression, and none had strong expression (Fig 1D). Our data do not support that expression levels of ROR1 are associated with aggressive disease. It is noteworthy that among female patients (n = 17), totally 76% (13/17) expressed ROR1, including 24% (4/17) with strong expression, while among male patients (n = 20) only 55% expressed ROR1, and none had strong ROR1 expression (Fig 1E). ROR1 expression was found to occur significantly more often in female than in male non-small cell lung cancer patients (p = 0.048). We detected weak or focal ROR1 expression in 4/37 (11%) of tissues judged to be adjacent to cancer.

**Table 1:** Drugs used and their IC_50_ values (μM) in NSCLC cells.

Drugs	MSTO-211H	PC9	NCI-H358	XLA-07	NCI-H1975	A549
Erlotinib	13.26 ± 1.38	(30.43 ± 1.82)×10^-3^	1.77 ± 0.10	> 20	5.41 ± 0.15	9.45 ± 0.55
Gefitinib	5.19 ± 0.40	(39.80 ± 4.98)×10^-3^	4.36 ± 0.68	20.67 ± 0.87	8.76 ± 0.47	10.69 ± 0.99

### Silencing of ROR1 with siRNA Induces Tumor-cell Death and Apoptosis

**Fig 2:**
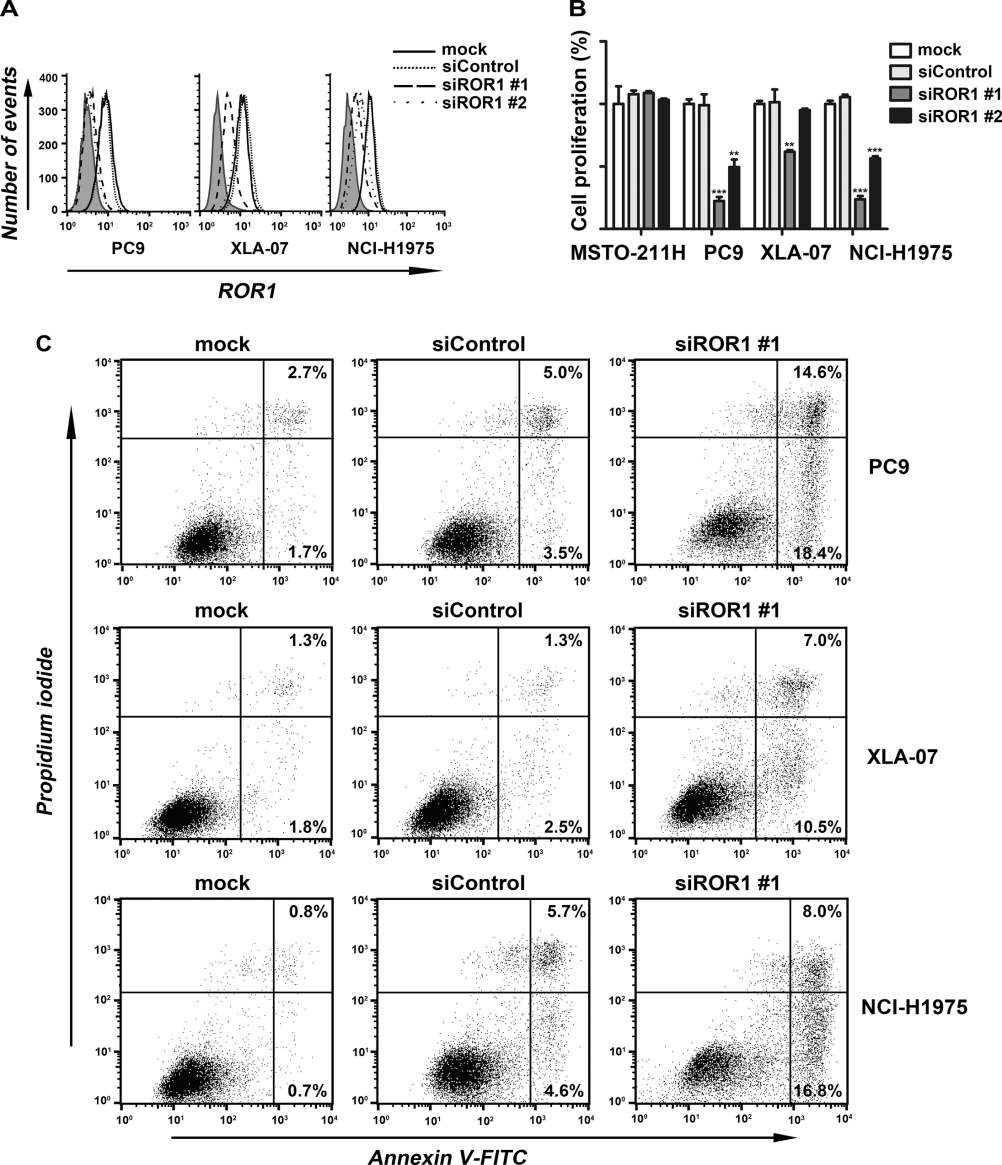
Blocking ROR1 via siRNA inhibited tumor cell growth and induced apoptosis. (A-C) PC9, XLA-07, and NCI-H1975 were treated with ROR1 siRNA (siROR1) or control siRNA (siControl) for 72 h and examined for ROR1 protein expression with chimeric rabbit/human anti-ROR1 monoclonal antibody R12 by flow cytometry (A), growth-inhibition by MTS assay (B) and apoptosis induction by Annexin-V/PI staining (C). The height of each bar in the graph B provides the mean number of viable cells that are representative of more than three independent experiments. *** p<0.001, ** p<0.01 by Student’s t test.

PC-9, XLA-07, and NCI-H1975 cell lines were transfected with pre-designed siROR1 and ROR1 expression was examined by flow cytometry at 72 h after transfection. Fig 2A shows siROR1-transfected NSCLC cells compared to cells transfected with the control, non-silencing siRNA (siControl) (considered to be 100%). The flow cytometry results demonstrated that the siROR1 silenced ROR1 expression by 50~90%.

The growth inhibitory effects of silencing by siROR1 were evaluated by MTS assays on PC-9, XLA-07, and NCI-H1975 cell lines. All cell lines silenced for ROR1 had reduced growth rates relative to those cells treated with the siControl (Fig 2B) (p<0.01, Student’s paired, two-tailed t-test). In contrast, no growth-inhibition (<10%) was induced in ROR1^−^ cell line MSTO-211H. Concomitantly, PC-9, XLA-07, and NCI-H1975 cell lines treated with siROR1 underwent apoptosis. Fig 2C demonstrates apoptosis induction in NSCLC cells following transfection with siRNA against ROR1. The level of apoptosis induction following siROR1 treatment varied between PC-9, XLA-07, and NCI-H1975 cell lines, but the level of apoptosis was markedly and significantly higher with siROR1 than apoptosis with the control non-silencing siRNA (18.4% vs 3.5%, 10.5% vs 2.5%, 16.8% vs 4.6%, respectively).

### ROR1 Activates PI3K/AKT/mTOR Signaling Pathway

**Fig 3:**
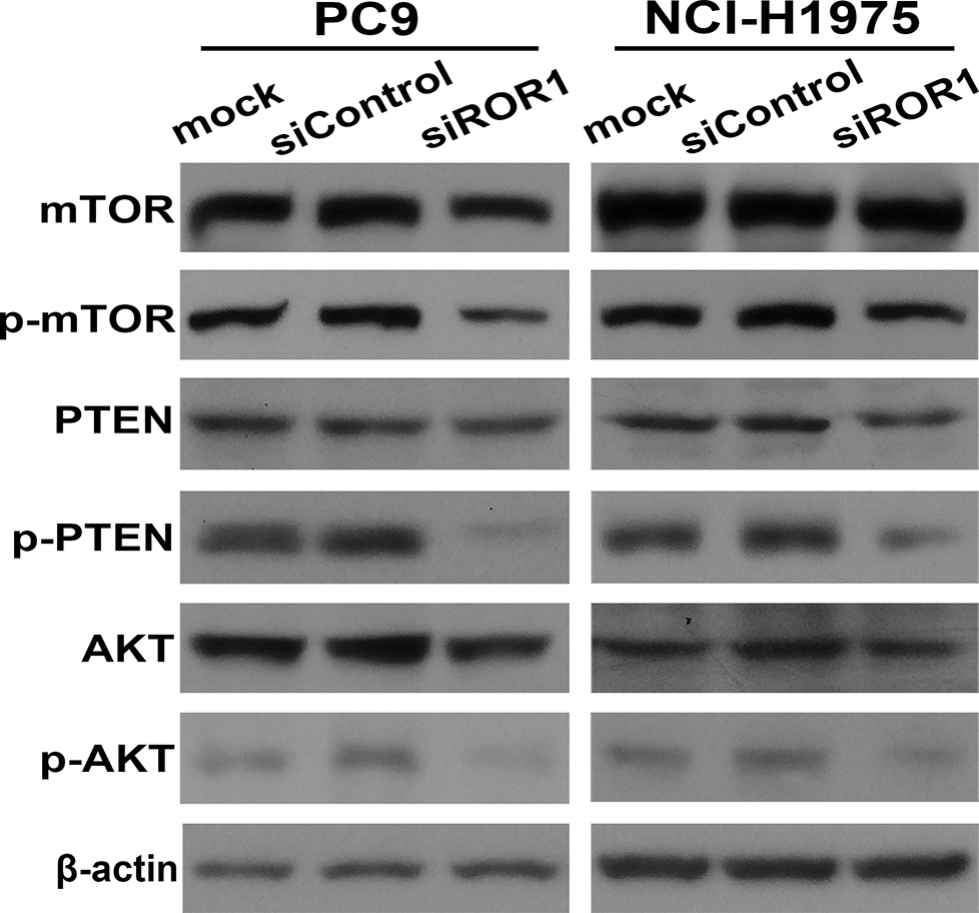
ROR1 activates the PI3K/AKT/mTOR signaling pathway. PC9 and NCI-H1975 were treated with ROR1 siRNA (siROR1) or control siRNA (siControl) for 72 h and examined for phosphorylated AKT at Ser-473 (p-AKT), phosphorylated mTOR at Ser-2448, and phosphorylated PTEN at Ser-380/Thr-382/383 (p-PTEN) by immunoblot analysis.

We also examined how ROR1 mediates survival signals in lung adenocarcinomas. The PI3K/AKT/mTOR pathway is altered in a variety of cancers including NSCLC. Aberrant activation of the PI3K/AKT/mTOR pathway is one of the mechanisms of acquired resistance to EGFR-TKI in patients with lung adenocarcinoma carrying EGFR activating mutations. We examined PC-9 and NCI-H1975 cells for phosphorylated AKT (p-AKT) at serine-473 (Ser-473), which is a feature of activated AKT. ROR1 knockdown decreased phosphorylation of both AKT (p-AKT Ser-473) and mTOR ((p-mTOR Ser-2448) in ROR1^+^ PC-9 and NCI-H1975 cells (Fig 3). siROR1 treatment also significantly reduced phosphorylation of PTEN (p-PTEN Ser-380/Thr-382/383), a negative regulator of PI3K/AKT signaling pathway. Collectively, these results indicate that activation of the PI3K/AKT/mTOR signaling pathway is associated with expression of ROR1 in PC-9 and NCI-H1975 cells.

## Discussion

Oncogenic driver mutations frequently occur in lung cancer and play roles in carcinogenesis. These mutations are usually associated with distinct clinical and histological features and are attractive targets for anticancer therapy. The majority of these oncogenic drivers are tyrosine kinases that are key regulators of cellular proliferation, growth, and survival. Constitutive activation of these kinases through mutations can be targeted using antibodies or inhibitory small molecules (TKI) directed at receptor tyrosine kinases. For example, Gefitinib and Erlotinib have provided clinical benefit in non-small cell lung cancer with activating EGFR mutations [35,36]. Despite the impressive high response rates to EGFR TKIs in EGFR-mutant NSCLC, prolonged clinical benefit is limited due to acquired drug resistance as previously described [37].

The expression or phosphorylation of ROR1 has shown good correlation with disease stages in patients with chronic lymphocytic leukemia (CLL) [38], ovarian cancer [22,24], pancreatic adenocarcinomas [22], and breast cancer [23]. Promising results in vitro and in vivo using ROR1 as a target to treat CLL, melanoma and breast cancer have been reported [21,23,25,39–43]. Although ROR1 expression in lung adenocarcinoma has been shown, the association of ROR1 expression with disease stages has not previously been studied extensively. We found about 65% of human lung adenocarcinoma tissues studied express ROR1. In contrast no or low expression of ROR1 was found in human squamous lung cancer (unpublished data) which indicates that ROR1 expression is more correlated with lung adenocarcinoma. In the present study, we did not see significant association of ROR1 expression intensity with disease stages. Possible reasons include: too few numbers of patients especially those with stages III and IV as well as those with scores 2 and 3 and the possibility that the phosphorylation and glycosylation level, but not the expression level of ROR1 may be correlated with aggressive lung adenocarcinoma. We cannot rule this out until the status of phosphorylation and glycosylation of ROR1 among patients is determined. We did observe that although the proportion of weakly or focally expressed ROR1 was much higher in stage I and II lung adenocarcinoma patients, moderately expressed ROR1 was more frequently found in stage III and IV patients. Therefore, larger stage III and IV patient populations should be included in future investigations. Karachaliouet al. [44] showed interesting data of different expression of ROR1 in lung adenocarcinoma patients with EGFR mutations sensitive or insensitive to Erlotinib which indicated that ROR1 expression is more associated with EGFR mutation status and that ROR1-directed therapies can enhance the efficacy of Erlotinib therapy. Data from our NSCLC cell lines study showed that ROR1 is expressed in cell lines NCI-H1975, NCI-H358, XLA-07, PC9, and A549 regardless of EGFR TKI sensitivity. In the following study it is more interesting to study the relationship between ROR1 expression and EGFR mutation status in larger population of lung adenocarcinoma patients. In our study we also observed that higher proportions of female patients expressed ROR1, and that strong ROR1 expression was only found in the women which is in accordance with the study that EGFR gene mutations were found to occur significantly more often in female than in male non-small cell lung cancer patients [45–47].

In order to clarify the growth-enhancing role of ROR1 in lung adenocarcinoma, we choose the TKI-resistant NCI-H1975 and XLA-07 cell lines and TKI-sensitive PC-9 cell line and found that blocking of ROR1 could significantly induce apoptosis and growth-inhibition of all those cell lines. This suggests the potential therapeutic value of both monoclonal antibodies against the extracellular part of RTKs and small molecules inhibiting the intracellular tyrosine kinase activity of RTKs, especially for lung adenocarcinomas with various TKI resistance mechanisms.

**Fig 4:**
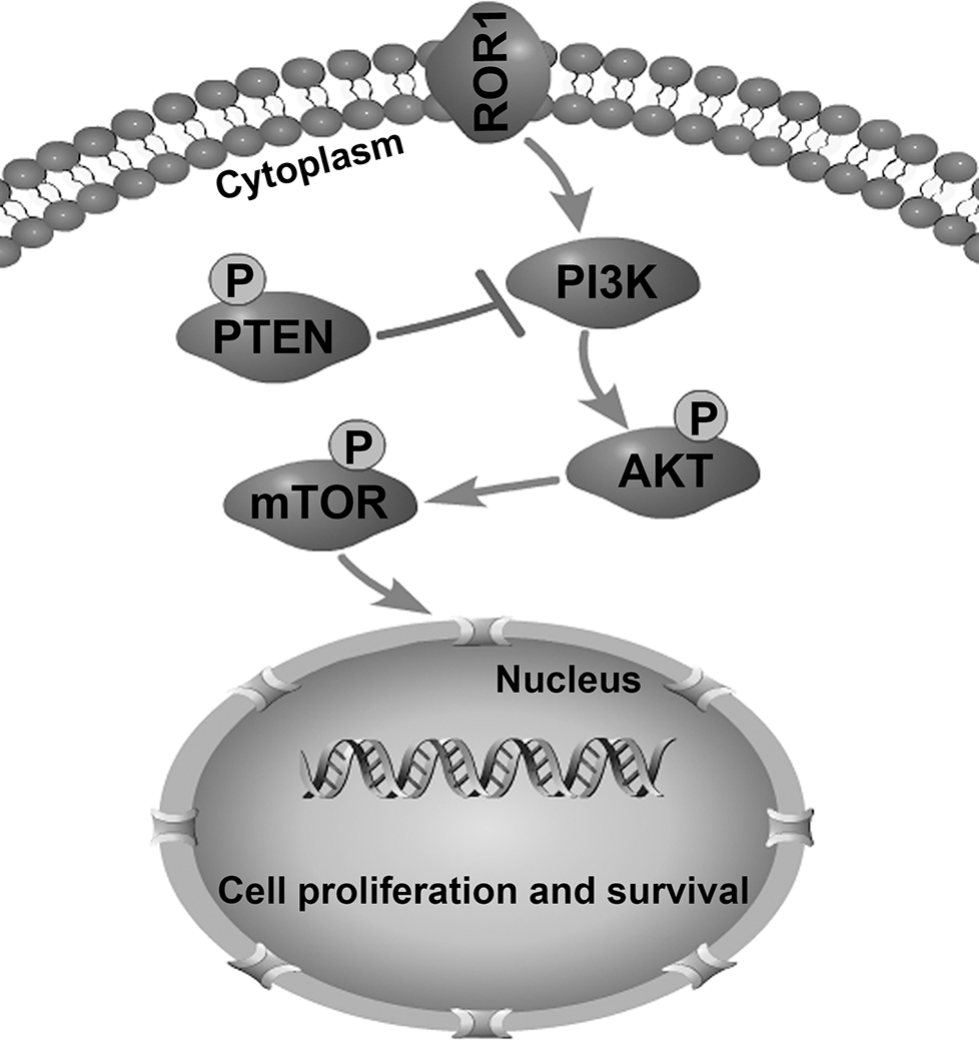
Proposed model of ROR1-mediated tumor cell survival and proliferation via PI3K/AKT/mTOR signaling pathway. Activation of ROR1 significantly enhanced phosphorylation of both AKT and mTOR, and deactivated PTEN, a negative regulator of PI3K/AKT.

Downstream signaling pathways of ROR1 are not fully known. The PI3K/AKT/mTOR pathway has been suggested to be involved as evaluated by hsRNA and mAb against ROR1 as recently published [23,48]. When we examined PI3K/AKT/mTOR signaling pathways to explain ROR1-mediated survival signals in lung adenocarcinomas, we found that silencing of ROR1 significantly decreased phosphorylation of both AKT and mTOR in ROR1^+^ PC-9 and NCI-H1975 cells. siROR1 treatment also significantly reduced phosphorylation of PTEN, a negative regulator of the PI3K/AKT signaling pathway. Based on our results we speculate that the PI3K/AKT/mTOR signaling pathway could be one of the ways that ROR1-mediated survival signaling occurs in lung adenocarcinoma (Fig 4). Additional studies are needed to clarify whether the critical molecules in the mTORC1, mTORC2, or both pathways play important roles in ROR1-mediated growth-enhancing signaling.

Until now the kinase function of ROR1 remains controversial. Studies revealed that ROR1 is an RTK-like pseudokinase and acts as MET and Src substrate [27,49–51]. Yamaguchi et al. [26] also showed that a cysteine-rich domain of the extracellular domain of ROR1 is required for association with EGFR sustaining EGFR-ERBB3-PI3K signaling. It will be important to clarify the relationship of MET and EGFR with ROR1 in lung adenocarcinoma patients. This could provide potential therapy regimens for lung cancer patients, especially those with TKI insensitivity and resistance.

In conclusion the present findings identify ROR1 as a potential oncogene target in lung adenocarcinomas. It is of particular interest that ROR1 inhibition appears to be effective for treatment of TKI-resistant lung adenocarcinomas with various resistance mechanisms. To date, very little is known about the functions of ROR1 and its role in human cancers and should be further investigated.
